# Analysis of Structural Flexibility of Damaged DNA Using Thiol-Tethered Oligonucleotide Duplexes

**DOI:** 10.1371/journal.pone.0117798

**Published:** 2015-02-13

**Authors:** Masashi Fujita, Shun Watanabe, Mariko Yoshizawa, Junpei Yamamoto, Shigenori Iwai

**Affiliations:** Division of Chemistry, Graduate School of Engineering Science, Osaka University, 1–3 Machikaneyama, Toyonaka, Osaka, 560–8531, Japan; Wake Forest University, UNITED STATES

## Abstract

Bent structures are formed in DNA by the binding of small molecules or proteins. We developed a chemical method to detect bent DNA structures. Oligonucleotide duplexes in which two mercaptoalkyl groups were attached to the positions facing each other across the major groove were prepared. When the duplex contained the cisplatin adduct, which was proved to induce static helix bending, interstrand disulfide bond formation under an oxygen atmosphere was detected by HPLC analyses, but not in the non-adducted duplex, when the two thiol-tethered nucleosides were separated by six base pairs. When the insert was five and seven base pairs, the disulfide bond was formed and was not formed, respectively, regardless of the cisplatin adduct formation. The same reaction was observed in the duplexes containing an abasic site analog and the (6–4) photoproduct. Compared with the cisplatin case, the disulfide bond formation was slower in these duplexes, but the reaction rate was nearly independent of the linker length. These results indicate that dynamic structural changes of the abasic site- and (6–4) photoproduct-containing duplexes could be detected by our method. It is strongly suggested that the UV-damaged DNA-binding protein, which specifically binds these duplexes and functions at the first step of global-genome nucleotide excision repair, recognizes the easily bendable nature of damaged DNA.

## Introduction

DNA bending is observed in various processes of life. There are two types of DNA bends. One is a smooth bend or curvature, such as the structure found in A-tract DNA [1]. The other is a sharp bend including kinks, in which the stacking interaction between the two base pairs at the junction is lost [2]. The latter DNA structure is formed by protein binding in the processes of transcription [3] and DNA repair [4–6]. The TATA-binding protein (TBP), which is a subunit of the general transcription factor TFIID, binds to the minor groove of the TATA box sequence located upstream of the transcription start site, and induces a sharp kink in the DNA [7, 8]. This preformed TBP–DNA complex structure is recognized by another transcription factor, TFIIB, in the initial steps of RNA polymerase recruitment for transcription in eukaryotic cells [9]. The DNA glycosylases responsible for base excision repair (BER) induce a helix kink, extrude the damaged base from the helix, and recognize its chemical structure by hydrogen bond formation at the substrate binding site, prior to the catalysis of the glycosidic bond cleavage. Among the BER enzymes, human 8-oxoguanine DNA glycosylase induces a very sharp bend at an angle of about 70°, as found in the crystal structure of its complex with the substrate duplex [10]. This enzyme also induces a large bend (about 80°) in its complex with undamaged DNA [11]. It was suggested that this bent structure is required to flip the guanine and 8-oxoguanine for the extrahelical inspection of the damage [12].

DNA duplexes are generally straight in the absence of protein binding. However, a sharp bend is induced when cisplatin (*cis*-diamminedichloroplatinum(II)), an anticancer drug, forms an intrastrand cross-link between the two N7 atoms of adjacent guanine bases, as determined by gel electrophoresis [13, 14], X-ray crystallography [15, 16], and NMR spectroscopy [17–19]. The reported bend angles ranged from 22° to 78°. In the crystal and NMR structures, the helix bending occurred toward the major groove; i.e., to the direction in which the major and minor grooves became narrow and wide, respectively, in the same manner as in protein-induced bending. The bend angle depends on the calculation method, and varies greatly even when the same structure is used [20]. Nevertheless, the cisplatin-adducted DNA is a rare example in which non-protein bound, bent DNA structures were detected by various methods. Proteins containing the high mobility group box recognize this unique structure of platinated DNA [21].

Another type of DNA that potentially has an intrinsic bend in its structure is a duplex containing an abasic site. The two base moieties flanking the abasic site can be brought close to each other, and thus the major groove width may become narrower. However, helix bending has not been observed in the structures of abasic site-containing duplexes determined by NMR [22–27]. Although the structures exhibited minor differences, these duplexes generally retained the B-form, with the unpaired base opposite the abasic site stacked in the helix and the abasic site sugar in a range of intrahelical to extrahelical conformations. For a duplex containing 3-hydroxy-2-(hydroxymethyl)tetrahydrofuran, as a stable analog of the abasic site, a bent structure with an angle of about 30° was reported [28]. However, hydrogen bonds between the opposite thymine and the 5’-flanking cytosine were found in this duplex, and these interactions probably stabilized the bent structure, because a very small bend angle (about 10°) was reported for another duplex containing the same abasic site analog in a different sequence context [29]. On the other hand, several studies have suggested that the abasic site increases the flexibility of the DNA duplex [30–33]. In one of them, the diffusion rate constants of oligonucleotide duplexes containing several types of DNA lesions, including the abasic site, were determined by NMR spectroscopy [30]. The diffusion rate obtained for a duplex containing the abasic site was intermediate between those for the undamaged duplex and the single-stranded oligonucleotide, implying that the loss of the base moiety considerably increased the flexibility of the duplex. The other studies were performed by molecular mechanics calculations and molecular dynamics simulations [31–33].

**Fig 1:**
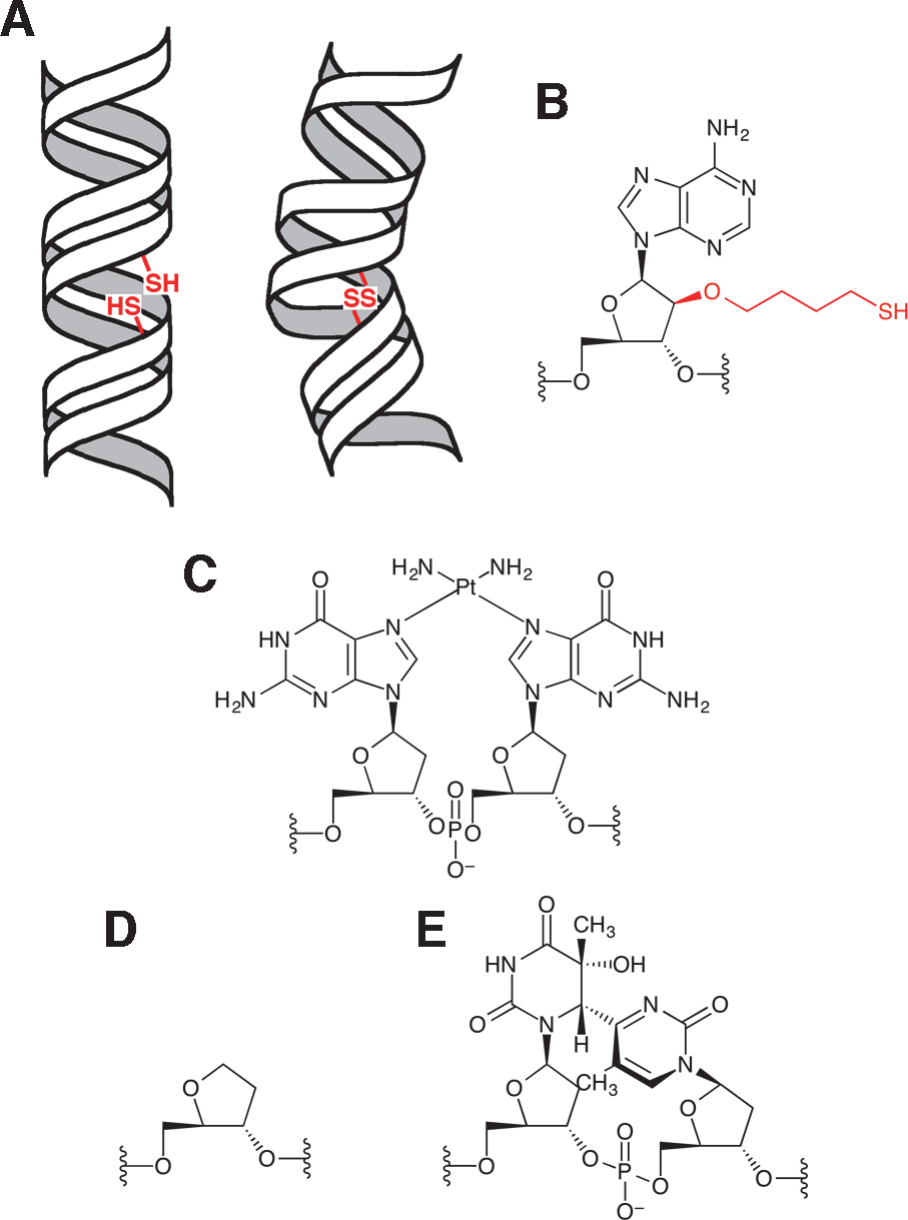
Disulfide bond formation dependent on the helix bending of DNA. (**A**) Schematic presentation of this study. Two mercaptoalkyl groups were attached to positions across the major groove of a duplex (left), and a disulfide bond was formed when helix bending occurred into the major groove (right). (**B**) The chemical structure of the thiol-tethered nucleoside. Another modified nucleoside bearing the 3-mercaptopropyl group was also used. (**C**–**E**) The chemical structures of the cisplatin adduct (**C**), the abasic site analog (**D**), and the (6–4) photoproduct (**E**).

To our knowledge, there are very few experimental methods to investigate the flexibility or bendability toward the major groove of a DNA duplex. To demonstrate such a property, rare bent conformations in the dynamic DNA structures should be shown, although the average conformations are usually obtained in structure studies. An electrophoretic method for the detection of the DNA flexibility was reported in 1994 [34], but the flexible locus in this study was an internal loop, which can be bent very easily in all directions. Recently, the dynamics between the two helices in RNA with a trinucleotide bulge were analyzed by using NMR residual dipolar couplings [35, 36], and a conformational ensemble of bulged DNA was determined by small-angle X-ray scattering interferometry, using gold nanocrystal probes [37]. However, the bent structure is the major conformation of bulged duplexes, and thus these methods are not suitable for the analysis of the bendability of abasic site-containing duplexes, which are expected to have an average structure similar to that of undamaged DNA. We recently collaborated with a research group in the field of electrochemistry to analyze the helix bending in damaged DNA [38], but static and dynamic bends could not be distinguished. In this article, we describe a method to detect helix bending in DNA, using the formation of a disulfide bond between two mercaptoalkyl groups attached to positions facing each other across the major groove in a duplex, as shown in Fig. 1. We applied this method to study the structures of DNA duplexes containing the abasic site analog and the pyrimidine(6–4)pyrimidone photoproduct ((6–4) photoproduct), and detected dynamic structural changes. Based on the results, the DNA recognition mechanism of the UV-damaged DNA-binding (UV-DDB) protein is discussed.

## Materials and Methods

### Synthesis of oligonucleotides bearing the (tritylthio)alkyl group

**Fig 2:**
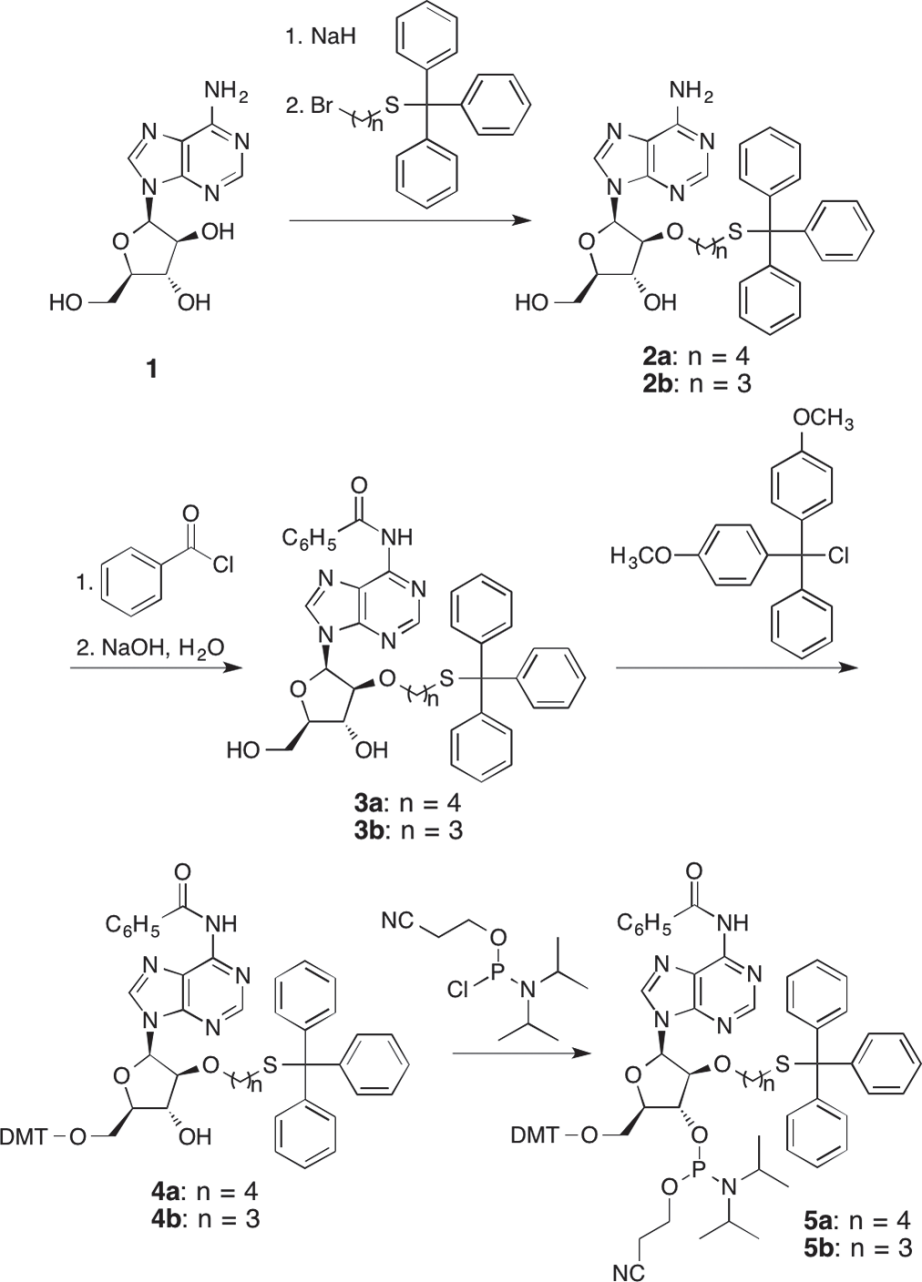
Synthesis of building blocks for the incorporation of a mercaptoalkyl group into oligonucleotides.

The compounds shown in Fig. 2 were synthesized as described in S1–S4 Protocols. Oligonucleotides were synthesized on an Applied Biosystems 3400 DNA synthesizer, using reagents purchased from Applied Biosystems and Glen Research. dSpacer CE Phosphoramidite (Glen Research) was used to synthesize the oligonucleotides containing the abasic site analog. The building blocks bearing the (tritylthio)alkyl group (**5a** and **5b**) were dissolved at a 0.1 M concentration in acetonitrile, and the reaction time was set to 20 min. After chain assembly, cleavage from the solid support and removal of the cyanoethyl group were performed simultaneously, by a treatment with 28% ammonia water (2 ml) at room temperature for 1 h, and the base protecting groups were removed by heating the ammoniac solution at 55°C for 6 h in a sealed vial. Oligonucleotides containing the (6–4) photoproduct were synthesized using the phosphoramidite building block described previously [39], using benzimidazolium triflate as an activator [40], and after cleavage from the support, the protecting groups for the base moieties were removed with ammonia water at room temperature for 3 h. The ammonia water was removed by evaporation, and the residue was dissolved in water (1 ml). The oligonucleotides were analyzed by HPLC on a Gilson gradient-type analytical system equipped with a Waters 2996 photodiode array detector, using a Waters μBondasphere C18 5μm 300A column (3.9 × 150 mm) at a flow rate of 1.0 ml/min with a linear gradient of 18.5–32% acetonitrile in 0.1 M triethylammonium acetate (TEAA, pH 7.0) over 20 min, at a column temperature of 60°C. After HPLC purification using a Waters μBondasphere C18 15μm 300A column (7.8 × 300 mm) at a flow rate of 2.0 ml/min, the eluate was concentrated on a rotary evaporator equipped with a vacuum pump, and TEAA was removed by co-evaporation with water. The amount of each oligonucleotide was determined from its absorbance at 260 nm using the molecular extinction coefficient calculated by the described method [41] (The UV absorption of the trityl group was ignored). Aliquots of the oligonucleotides were analyzed by matrix-assisted laser desorption/ionization time-of-flight (MALDI-TOF) mass spectrometry. The cisplatin adduct was formed by incubating the oligonucleotide (360 nmol) with *cis*-diamminedichloroplatinum(II) (700 nmol) in water (300 μl) at 16°C for 3 days, and the product was purified by HPLC, in a manner similar to that described above.

### Analysis of the interstrand disulfide-bond formation

The oligonucleotide bearing the (tritylthio)alkyl group (0.5 A_260_ units) was dissolved in 10 mM TEAA (50 μl), and was mixed with 0.1 M silver nitrate (7.5 μl). After 30 min, 0.1 M dithiothreitol (DTT, 10 μl) was added, and the mixture was allowed to react for 15 min. The precipitate was separated by centrifugation at 15,000 rpm for 15 min, washed with 10 mM TEAA (50 μl), and separated again by centrifugation at 15,000 rpm for 5 min. The supernatant and the wash solution were combined and applied to an illustra NAP 5 column (GE Healthcare). The deprotected oligonucleotide was eluted with water (950 μl), and then 0.1 M DTT (10 μl) was added. The solutions of the two strands (0.5 nmol per each strand) were mixed, and the mixture was dried in a Speed Vac concentrator. The residue was dissolved in 10 mM sodium phosphate (pH 7.0, 40 μl), and after heating at 60°C for 2 min and cooling gradually to room temperature, the solution was applied to an illustra NAP 5 column equilibrated with an oxygen-bubbled buffer, containing 100 mM NaCl and 10 mM sodium phosphate (pH 7.0). Elution was performed with the same buffer (700 μl), and the eluate was placed in a 4-ml vial (clear silanized glass, National Scientific) with a rubber septum. Oxygen was bubbled through the solution for 2 min, and then an oxygen balloon was attached to the vial. After incubations at 23°C for the reaction times indicated in the figures, HPLC analyses were performed on a Gilson gradient-type analytical system equipped with a Waters 2996 photodiode array detector, using a Waters μBondasphere C18 5μm 300A column (3.9 × 150 mm) at a flow rate of 1.0 ml/min with a linear gradient of 5.0–18.5% acetonitrile in 0.1 M TEAA over 20 min, at a column temperature of 60°C. For the analyses of the (6–4) photoproduct-containing duplexes, a Waters XBridge C18 5μm column (4.6 × 150 mm) was used with a linear gradient of 7–16% acetonitrile. Since the retention times slightly changed in every injection, with deviations within 1 min, the x-axis in each chromatogram was adjusted by comparing the UV absorption spectra of the peaks and deleting several data points between 0 and 5 min. The DTT treatment was performed by mixing the sample solution (100 μl) with 0.1 M DTT (50 μl), followed by an incubation at 23°C for 12 h. The experiments were repeated three times to confirm the reproducibility.

## Results

### Synthesis of oligonucleotides bearing a mercaptoalkyl group

We intended to develop a method to detect the helix bending of DNA by the formation of a disulfide bond, as shown in Fig. 1A, using the cisplatin-adducted duplex (Fig. 1C), which was proved to be bent by several methods [13–19], as a model system. Since the adduct formation bends the helix toward the direction in which the major groove becomes narrow [15–19], we searched for the positions in the canonical B-form DNA that would become closer when this type of structural change occurred, and found that the 2’ upper position could be a candidate. If a mercaptoalkyl group was attached to this position, then this side chain should point toward the complementary strand across the major groove. The 2’ lower position might have a problem of steric hindrance with the 3’ flanking nucleoside, and the other sugar protons were not located in the major groove. Therefore, we decided to prepare duplexes containing 2-*O*-(4-mercaptobutyl)arabinofuranose as a sugar moiety (Fig. 1B), at a single site in each strand. It should be noted that the sugar conformation of arabinonucleoside is the same as that of 2’-deoxyribonucleoside in a duplex [42].

Manoharan et al. reported the synthesis of oligonucleotides containing the 6-(tritylthio)hexyl group attached to the 2’ hydroxyl function of adenosine [43, 44]. To obtain this modified nucleoside, they preferentially alkylated the 2’-hydroxyl function of unprotected adenosine, by the abstraction of the 2’-OH proton with sodium hydride followed by the reaction with bromoalkane. We applied this method to the alkylation of 9-(β-D-arabinofuranosyl)adenine (**1**), as shown in Fig. 2. The reaction occurred preferentially at the 2’ position of this arabinose-containing nucleoside analog, in the same manner as the ribonucleoside. As a byproduct, the 5’-*O*-alkyl derivative was obtained, while the side reaction was 3’-*O*-alkylation in the case of the ribose [43, 44]. These alkylation positions were easily determined from the cross-peaks between the OH and CH protons at the C2’, C3’, and C5’ positions in the COSY spectra. Since the 2’-*O*- and 5’-*O*-alkylation products could not be separated on a silica gel column, the desired product was isolated by reversed-phase chromatography, although fractions containing both of the products remained. The obtained compound, 9-[2-*O*-[4-(tritylthio)butyl]-β-D-arabinofuranosyl]adenine (**2a**), was derivatized to a phosphoramidite building block (**5a**) in three steps, as shown in Fig. 2, and oligonucleotides containing this modified nucleoside were synthesized. To avoid the formation of non-specific disulfide bonds, the trityl group, which is stable during the oligonucleotide deprotection steps and can be removed selectively with silver nitrate, was kept on the mercapto function until the oligonucleotides were utilized.

### Disulfide bond formation in cisplatin-adducted duplexes

**Fig 3:**
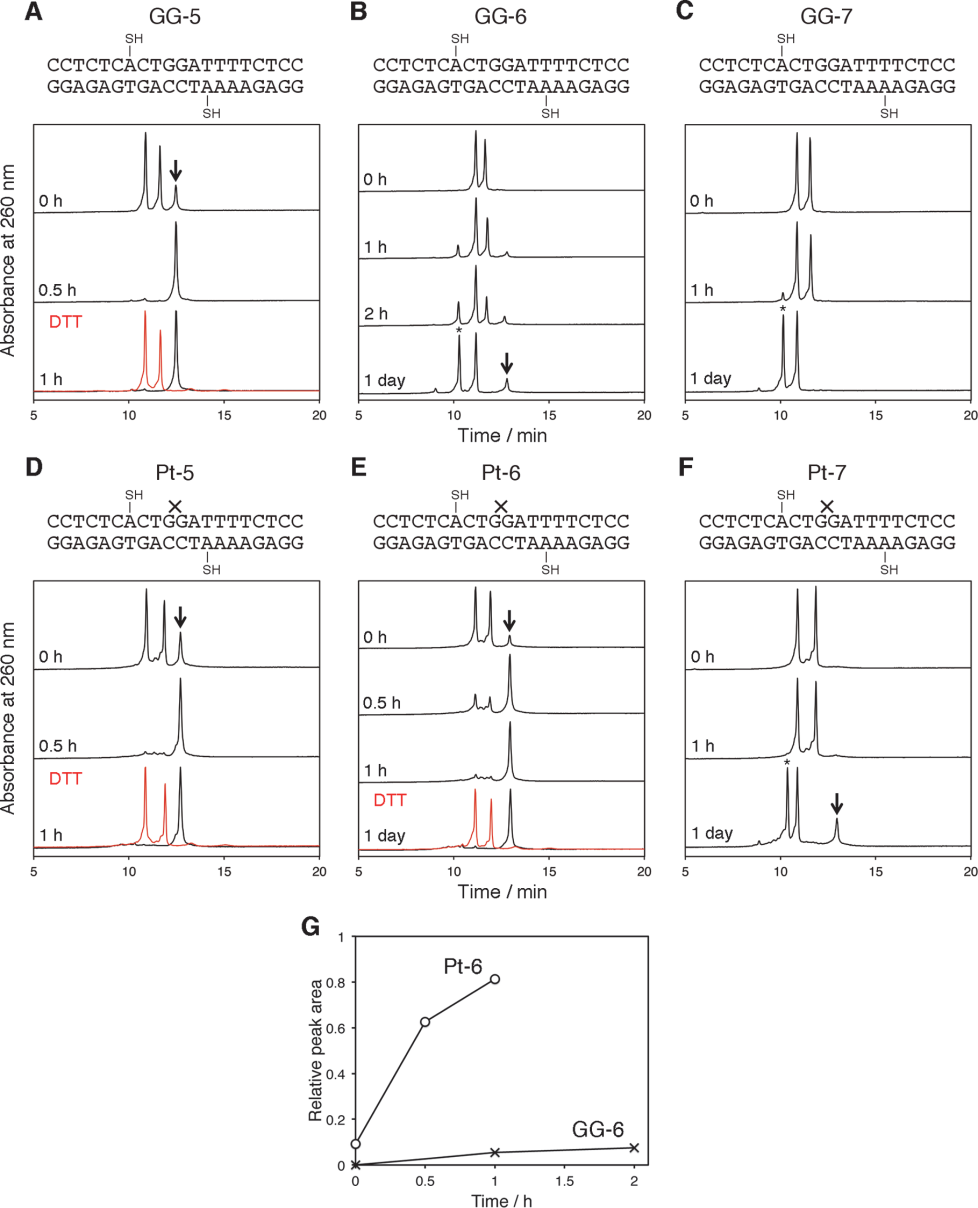
HPLC analysis of disulfide bond formation in duplexes containing the cisplatin adduct. (**A**–**F**) Chromatograms of the duplexes, in which × represents cisplatin, after the reactions for the indicated length of time. The y-axis of each chromatogram was normalized. The cross-linked products and the sulfinic acid-containing oligonucleotides are indicated by an arrow and an asterisk, respectively, and the results of the DTT treatment of the products are shown in red. (**G**) Comparison of the product formation between GG-6 and Pt-6. The product peak areas in panels **B** and **E** were quantified.

**Fig 4:**
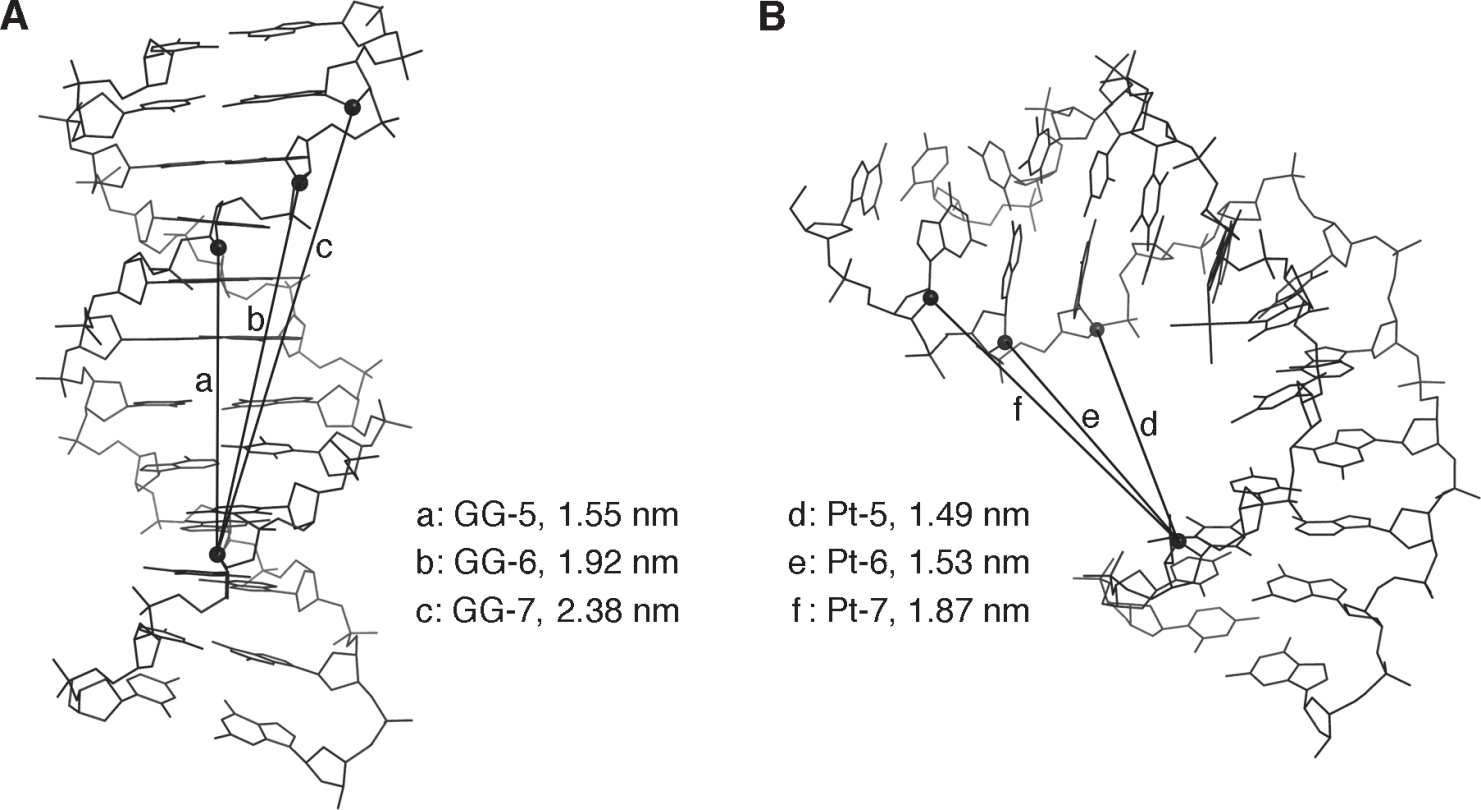
Structures of the B-form (A) and cisplatin-induced bent (B) DNA duplexes. The duplexes with the PDB codes of 2K0V and 1A84 are shown. The distances between the C2’ atoms of the modified nucleosides were calculated using the PyMOL 1.7.1 software.

The duplexes used for the analysis of the structure-dependent disulfide bond formation are shown in Fig. 3. Considering that the cisplatin adduct should be prepared at the GG site after the oligonucleotide synthesis, the sequence of the 20-base-pair (bp) duplex was designed to contain guanine only at the central GG in the top strand, and the modification with the 4-mercaptobutyl group in the cisplatin-containing strand was placed at the 7th nucleoside from the 5’ end. When a line parallel to the helix axis was drawn from the sugar moiety of this modified nucleoside across the major groove in B-form DNA, it reached the nucleoside that base-paired with the 6th nucleoside in the 3’ direction from the first modification site (Fig. 4A). Therefore, duplexes with a 5 bp insert between the two mercaptobutyl-attached nucleoside analogs were designed (GG-5 and Pt-5 in Fig. 3). Since unwinding at the cisplatin adduct site was reported in the NMR structure [18], duplexes with longer inserts in the same sequence context (GG-6, Pt-6, GG-7, and Pt-7) were also designed, to adjust the phase of the helix and to change the distance between the SH groups.

After the preparation of the oligonucleotides containing the 4-(tritylthio)butyl group with and without cisplatin, the following procedures were performed prior to the disulfide bond formation: deprotection of the mercapto function with silver nitrate, removal of the silver nitrate by gel filtration, duplex formation in the presence of DTT, and removal of the DTT by gel filtration. The second gel filtration step was the starting point of the reaction for the formation of the interstrand disulfide bond, which was analyzed by reversed-phase HPLC under heat-denaturing conditions. We expected that the product with an interstrand cross-link would be detected as a new peak, which would be converted to the two peaks of the original SH-containing oligonucleotides by the addition of a reducing agent. In our preliminary experiments, solutions of the duplexes were prepared, as described above, and aliquots were analyzed by HPLC at one day intervals. A new peak, which disappeared by the addition of DTT, was detected for GG-5, Pt-5, and Pt-6. However, the reaction occurred very slowly, and the results could not be reproduced accurately. We focused on the dissolved oxygen (DO), which is required for disulfide bond formation, and tried to accelerate the reaction by increasing its concentration. Measurements of the DO concentration in the solution revealed that a high DO concentration (about 400% of DO at ambient atmospheric pressure) could be maintained by bubbling oxygen gas through the solution and attaching an oxygen balloon to the sealed vial. Under this oxygen atmosphere, both the reaction rate and its reproducibility were improved to great extents.

In the experiments using the duplexes containing the 5 bp insert (GG-5 and Pt-5), a new peak appeared immediately after the removal of DTT from the solution, and the starting materials were completely converted to this product within 30 min (Fig. 3A and D). The product peaks were reverted to the original peaks by the addition of DTT. This result demonstrated that the reaction that occurred in these duplexes was the interstrand disulfide bond formation. In the experiments using the duplexes containing one more base pair between the thiol-tethered arabinonucleosides (GG-6 and Pt-6), different results were obtained in the presence and absence of cisplatin (Fig. 3B and E). The results with Pt-6 were similar to those with GG-5 and Pt-5, but the disulfide bond formation in GG-6 was extremely slow (Fig. 3G). Instead, one of the original peaks, which was identified as the top strand of GG-6, shifted to another peak with a shorter retention time, as indicated by an asterisk in Fig. 3B, while the other peak did not change. This product was isolated and analyzed by MALDI-TOF mass spectrometry, and the obtained *m*/*z* value (6106.08) was larger by 32 than that calculated for the top strand of GG-6 (6074.03). This result suggested that the product contained sulfinic acid (-SO_2_H), which was derived from the oxidation of the mercapto function. When the mercaptobutyl groups were separated by the 7 bp insert (GG-7 and Pt-7), this oxidation product was primarily formed, and the interstrand disulfide bond was not formed efficiently even in the presence of the cisplatin adduct (Fig. 3C and F).

### Analysis of the abasic site- and (6–4) photoproduct-containing duplexes

**Fig 5:**
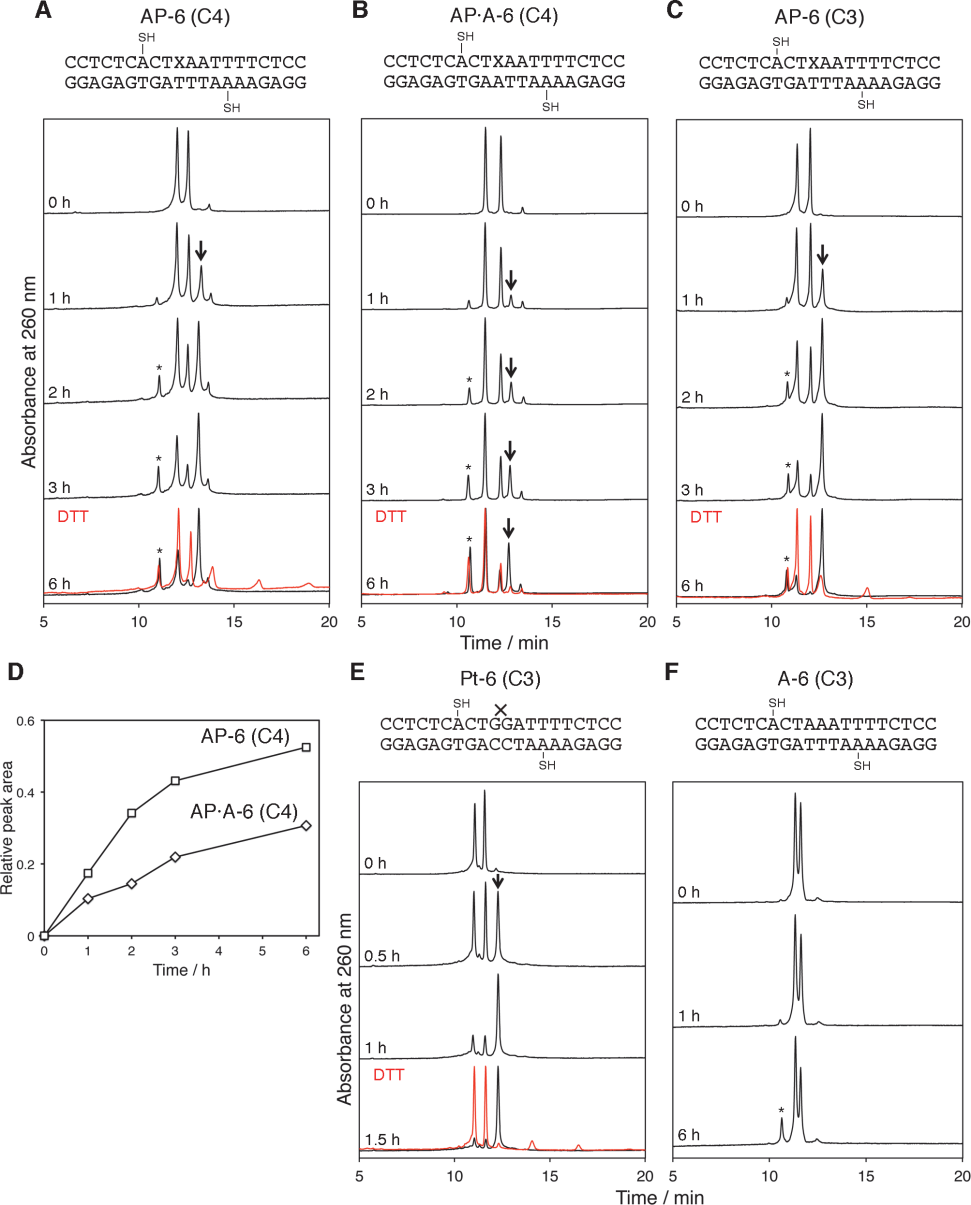
HPLC analysis of disulfide bond formation in duplexes containing the abasic site analog. (**A**, **B**, **C**, **E**, and **F**) Chromatograms of the duplexes, in which X represents the abasic site analog, after the reactions for the indicated length of time. C4 and C3 represent the 4-mercaptobutyl and 3-mercaptopropyl groups, respectively. The y-axis of each chromatogram was normalized. The cross-linked products and the sulfinic acid-containing oligonucleotides are indicated by an arrow and an asterisk, respectively, and the results of the DTT treatment of the products are shown in red. (**D**) Comparison of the product formation between AP-6 (C4) and AP·A-6 (C4). The product peak areas in panels **A** and **B** were quantified.

The same experiments were performed using duplexes containing the stable abasic site analog, 3-hydroxy-2-(hydroxymethyl)tetrahydrofuran (Fig. 1D). As shown in Fig. 5A, two product peaks were detected in the analyses at 1 h intervals. The product that yielded a peak with a longer retention time (about 13 min, indicated by an arrow) was generated at a higher rate than the product that eluted faster (at about 11 min). This slow-eluting compound, which yielded the largest peak when one of the starting oligonucleotides was exhausted, was assigned to the cross-linked duplex containing the interstrand disulfide bond, because its peak was reverted to the peaks of the starting materials by the addition of DTT. The other product, indicated by an asterisk in Fig. 5A, was the sulfinic acid-containing oligonucleotide derived from the top strand, which was obtained similarly with GG-6, GG-7, and Pt-7 (Fig. 3). At 6 h after the DTT removal, the top strand was consumed almost completely to yield the two products, while the bottom strand remained. The results of the experiments using duplexes with different numbers of inserted base pairs (AP-5 and AP-7, S1A and B Fig.) were similar to those obtained with Pt-5 and Pt-7 (Fig. 3D and F), respectively, although the disulfide bond formation in AP-5 was slower than that in Pt-5.

An NMR study revealed that the local sequence context influenced the structure of the abasic site-containing DNA [27]. The conformation around the abasic site was more perturbed when the opposite base was a pyrimidine than a purine. Therefore, we tested a duplex containing A opposite the abasic site (Fig. 5B). The results were similar to those of the duplex containing T, but the formation of the cross-linked product was slower (Fig. 5D).

To obtain further information, we synthesized a building block of 9-(β-D-arabinofuranosyl)adenine bearing the 3-(tritylthio)propyl group (**5b**), and incorporated it into oligonucleotides. The duplex consisting of these oligonucleotides contained the 3-mercaptopropyl group, which was shorter by one carbon unit than the above-mentioned 4-mercaptobutyl group, in each strand. The results (Fig. 5C) were very similar to those obtained with the 4-mercaptobutyl counterpart (Fig. 5A). Experiments using the cisplatin-adducted duplex bearing the 3-mercaptopropyl groups were also performed (Fig. 5E). It should be noted that the length of the alkyl group affected the rate of the disulfide bond formation, in the case of the cisplatin adduct (Figs. 3E and 5E). Although the sequences were different, a similar dependency of the reaction rate on the alkyl chain length was observed for the 5 and 6 bp-inserted duplexes without the cisplatin adduct or the abasic site analog (Fig. 3A and S2A Fig. for 5 bp, and Figs. 3B and 5F for 6 bp), while there was no difference between the 7 bp-inserted duplexes that did not yield the desired product peak (Fig. 3C and S2B Fig.). Therefore, the result that similar reaction rates were observed for the abasic site, regardless of the alkyl chain lengths, was very distinguishing.

**Fig 6:**
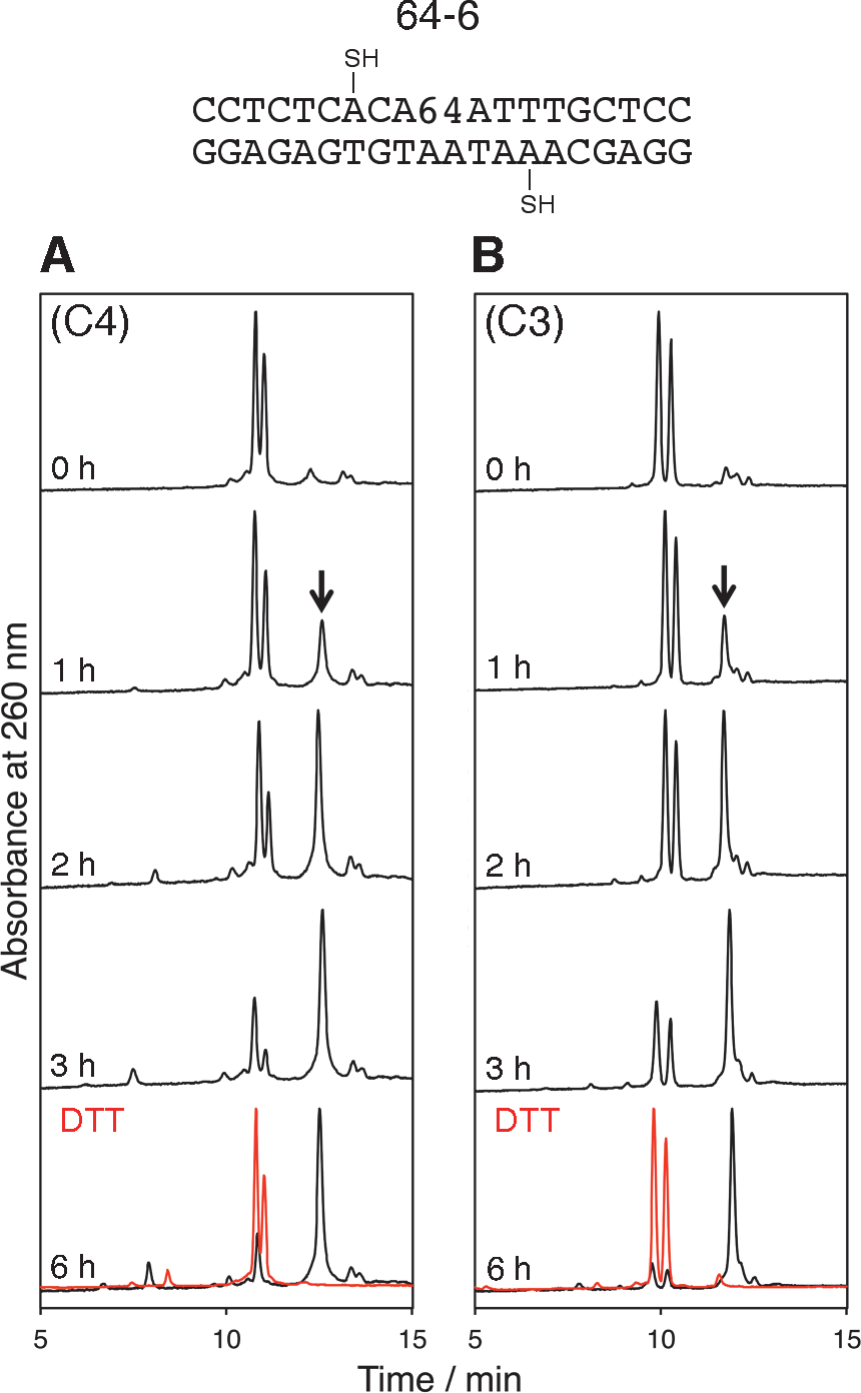
HPLC analysis of disulfide bond formation in duplexes containing the (6–4) photoproduct. C4 and C3 represent the 4-mercaptobutyl and 3-mercaptopropyl groups, respectively. The y-axis of each chromatogram was normalized. The cross-linked products are indicated by an arrow, and the results of the DTT treatment of the products are shown in red.

**Fig 7:**
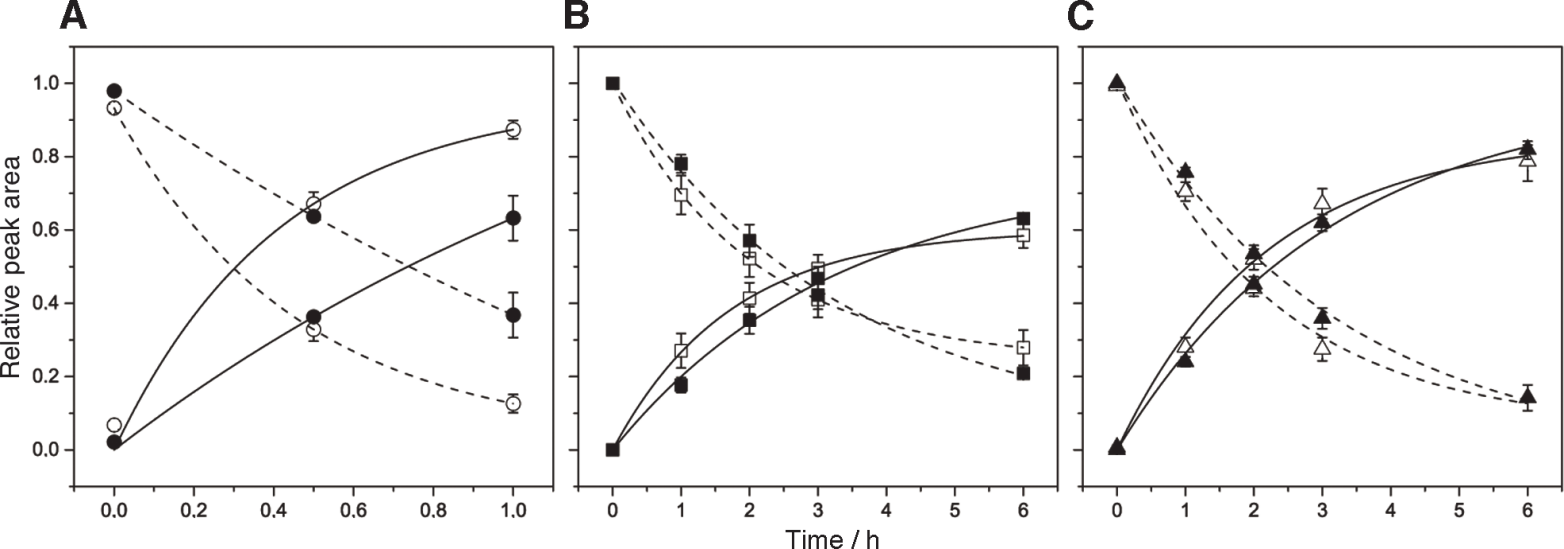
Effect of the linker length on the rate of the disulfide bond formation. (**A**) Pt-6 (C4) (open circles) and Pt-6 (C3) (filled circles); (**B**) AP-6 (C4) (open squares) and AP-6 (C3) (filled squares); (**C**) 64–6 (C4) (open triangles) and 64–6 (C3) (filled triangles). The dashed and continuous lines represent the decrease of the starting materials and the increase of the cross-linked products, respectively, and the standard errors are shown. The curve fitting was performed with the Origin 9.1 software, and the kinetic constants for the product formation are listed in Table 1.

Our previous study showed that the human UV-DDB protein recognizes DNA containing the abasic site analog (Fig. 1D) and the (6–4) photoproduct (Fig. 1E), which is one of the major UV-induced lesions, in the same manner [45], and we have been interested in the structural similarity between these two types of damaged DNA. Therefore, the structural properties of the duplex containing the (6–4) photoproduct were investigated. The sequence of the duplex was slightly changed from that used for the abasic site analog, as shown in Fig. 6, to avoid a long stretch of A, and the HPLC column was also changed to separate the two strands constituting the 64–6 duplex. As shown in Fig. 6A and B, and S1C and D Fig., the results were almost the same as those obtained with the duplexes containing the abasic site analog (Fig. 5A and C, S1A and B Fig.). As observed for the abasic site analog, there was no distinct difference between the duplexes containing the butyl and propyl linkers (Fig. 7).

**Table 1:** Kinetic constants for the product formation.

Duplex	*k* (h^–1^)
Pt-6 (C4)	2.399
Pt-6 (C3)	0.595
AP-6 (C4)	0.585
AP-6 (C3)	0.307
64–6 (C4)	0.459
64–6 (C3)	0.315

## Discussion

In this study, we first developed a chemical method to detect the helix bending of DNA. The interstrand disulfide bond formation was successfully utilized to detect the bent structure of the oligonucleotide duplexes. When the 4-mercaptobutyl groups were separated by a 6 bp insert across the major groove (GG-6 and Pt-6 in Fig. 3), a disulfide bond was formed only when the duplex contained the cisplatin adduct. There are two factors that should be taken into account. One is the phase of the helix, and the other is the distance across the major groove. Although the cisplatin adduct formation reportedly unwinds the helix [18], its angle (25°) is smaller than the rotation between the adjacent base pairs in B-form DNA (36°), and the mercaptoalkyl group is flexible. Therefore, the distance between the C2’ positions of the two alkylated arabinonucleosides is important in the present method. Correspondingly, the results obtained for the duplexes containing the 5 bp and 7 bp inserts indicated that the distances between the two modified nucleosides in these duplexes were simply too short and too long, respectively, to detect the structural difference. The B-form and cisplatin-adducted duplex structures and the calculated distances between the C2’ atoms of the modified nucleosides are shown in Fig. 4. The bend angle of the duplex containing the cisplatin adduct (Fig. 4B) is reportedly 78° [18]. The distance between the C2’ and sulfur atoms in the modified nucleoside is 0.76 nm when the 4-mercaptobutyl group is stretched. Although there may be structural fluctuations, the above discussion is supported by this value and the distances shown in Fig. 4.

For the duplexes in which the disulfide bond was not formed (GG-6, GG-7, and Pt-7), another type of product was detected, and the mass analysis revealed that the mercapto function in the top strand was oxidized to sulfinic acid. On the other hand, the mercapto function in the bottom strand was not oxidized. This observation suggested that the oxidation to the sulfinic acid depended on the sequence around the mercaptoalkyl group. The modified nucleoside in the top strand was located between two C·G pairs, while that in the bottom strand was not flanked by either a C·G or G·C pair. Since the guanine base has the lowest oxidation potential among the four nucleobases [46], it is presumed that the mercapto function in the top strand was oxidized more easily due to the presence of the neighboring guanine bases, which could be oxidized to radical cations. We confirmed that the oxidation pattern was changed, by altering the sequences around the modified nucleosides (S3 Fig.).

The same types of experiments were performed with duplexes containing a stable abasic site analog, and the results were comparable to those obtained for the cisplatin adducted duplexes, although the rate of disulfide bond formation was slower. When the length of the alkyl groups was changed from four carbons to three, very similar results were obtained for the duplexes containing the abasic site analog (Fig. 5A and C, and Fig. 7B), whereas in the case of the cisplatin adduct, the reaction rate was reduced by shortening the alkyl chains (Figs. 3E, 5E, and 7A, Table 1). Recently, Chiba et al. reported that their electrochemical analysis demonstrated the helix bending in duplexes containing several types of DNA lesions including the same abasic site analog [38]. Their study supports the idea that the disulfide bond was formed depending on the helix bending in our study. In addition, we determined the bent structure of the cross-linked duplex by NMR, although the results will be published elsewhere. The finding that the duplexes with the butyl and propyl linkers yielded very similar results (Fig. 7B) indicated a dynamic structural change in the abasic site-containing duplex, as contrasted with the static bending observed for the cisplatin-adducted duplex in which the linker length affected the rate of the disulfide bond formation (Fig. 7A, Table 1). In other words, the duplex containing an abasic site dynamically fluctuates between the straight and bent structures, although the majority of the duplexes are in the straight form, and the bent conformation was captured by the disulfide bond formation.

We further applied our method to the duplexes containing the (6–4) photoproduct, which is formed between two adjacent pyrimidine bases by exposure to UV light. Two inconsistent structures were reported for the duplex containing the (6–4) photoproduct. Kim and Choi reported an NMR structure with a large helix bend [47], while an unrestrained molecular dynamics analysis [48] and our study using fluorescence resonance energy transfer [49], which were subsequently conducted to verify the NMR structure, revealed significantly smaller or no bending. In the present study, the results obtained with the duplexes containing the (6–4) photoproduct were similar to those acquired with the duplexes containing the abasic site analog, rather than those containing the cisplatin adduct (Fig. 7, Table 1). This observation supports the unbent structure as the average conformation of the (6–4) photoproduct-containing duplex, and indicates that the photoproduct formation makes the duplex flexible. The positional shift of the 3’ component of the (6–4) photoproduct, which is linked orthogonally to the 5’ base (Fig. 1E), could be important for the similarity to the abasic site. The UV-DDB protein recognizes the UV-induced lesions in DNA at the first step of the global-genome nucleotide excision repair pathway in eukaryotic cells. Although the DNA recognition mechanism of the UV-DDB protein has not been elucidated, this protein has high affinity for the duplexes containing the (6–4) photoproduct and the abasic site analog, while its affinity for the cisplatin-adducted duplex is much lower [45], and large helix bending was found in the protein–DNA complex [45, 50]. Since no interaction was found between the damaged base and the protein in the crystal structure of the complex [50], some physical property of the damaged DNA must be recognized by this protein. The results in this study strongly suggest that the UV-DDB protein searches for the structural flexibility induced by the DNA damage, and specifically binds to the site where helix bending occurs easily.

We developed a chemical method to detect the static and dynamic bending of DNA. A unique feature of this method is that the dynamic structural change or the flexibility of DNA can be determined by capturing the rare bent conformation of the helix. Using this method, we detected the structural flexibility of the duplexes containing the abasic site analog and the (6–4) photoproduct, and proposed the DNA recognition mechanism of the UV-DDB protein. Since bent DNA structures have been found in many protein–DNA complexes, our present study will contribute toward the elucidation of the molecular mechanisms of various biological processes involving protein–DNA interactions.

## Supporting Information

S1 Fig HPLC analysis of disulfide bond formation in duplexes containing the abasic site analog (A and B) and the (6–4) photoproduct (C and D) with the 4-mercaptobutyl groups. The y-axis of each chromatogram was normalized. The oligonucleotides containing sulfinic acid are indicated by an asterisk, and the results of the DTT treatment are shown in red.
(TIF)

S2 Fig HPLC analysis of disulfide bond formation in duplexes containing 2’-deoxyadenosine instead of the abasic site analog with the 3-mercaptopropyl groups. The y-axis of each chromatogram was normalized. The cross-linked products and the sulfinic acid-containing oligonucleotides are indicated by an arrow and an asterisk, respectively, and the result of the DTT treatment is shown in red.
(TIF)

S3 Fig HPLC analysis of the oxidation of the mercapto group in a different sequence. The peaks of the starting oligonucleotides were assigned by co-injection. The product had the same UV absorption spectrum as the bottom strand.
(TIF)

S1 Protocol Synthesis of 9-[2-*O*-[4-(tritylthio)butyl]-β-D-arabinofuranosyl]adenine (2a) and 9-[2-*O*-[3-(tritylthio)propyl]-β-D-arabinofuranosyl]adenine (2b). (DOCX)

S2 Protocol Synthesis of 6-*N*-benzoyl-9-[2-*O*-[4-(tritylthio)butyl]-β-D-arabinofuranosyl]adenine (3a) and 6-*N*-benzoyl-9-[2-*O*-[3-(tritylthio)propyl]-β-D-arabinofuranosyl]adenine (3b). (DOCX)

S3 Protocol Synthesis of 6-*N*-benzoyl-9-[5-*O*-(4,4'-dimethoxytrityl)-2-*O*-[4-(tritylthio)butyl]-β-D-arabinofuranosyl]adenine (4a) and 6-*N*-benzoyl-9-[5-*O*-(4,4'-dimethoxytrityl)-2-*O*-[3-(tritylthio)propyl]-β-D-arabinofuranosyl]adenine (4b). (DOCX)

S4 Protocol Synthesis of 6-*N*-benzoyl-9-[5-*O*-(4,4'-dimethoxytrityl)-3-*O*-[[bis(1-methylethyl)amino](2-cyanoethoxy)phosphino]-2-*O*-[4-(tritylthio)butyl]-β-D-arabinofuranosyl]adenine (5a) and 6-*N*-benzoyl-9-[5-*O*-(4,4'-dimethoxytrityl)-3-*O*-[[bis(1-methylethyl)amino](2-cyanoethoxy)phosphino]-2-*O*-[3-(tritylthio)propyl]-β-D-arabinofuranosyl]adenine (5b). (DOCX)
