# Correction: Analysis of Structural Flexibility of Damaged DNA Using Thiol-Tethered Oligonucleotide Duplexes

**DOI:** 10.1371/journal.pone.0123038

**Published:** 2015-03-30

**Authors:** The PLOS ONE Staff

The text from the Funding section is incorrectly included in the Competing Interests section. The correct Competing Interests information is as follows: The authors have declared that no competing interests exist. The publisher apologizes for this error, which was introduced during the typesetting process.

## References

[1] Fujita M, Watanabe S, Yoshizawa M, Yamamoto J, Iwai S (2015) Analysis of Structural Flexibility of Damaged DNA Using Thiol-Tethered Oligonucleotide Duplexes. PLoS ONE 10(2): e0117798 doi:10.1371/journal.pone.0117798 25679955 10.1371/journal.pone.0117798 PMC4332495

