# Supplementary figures and images for: Analysis of Structural Flexibility of Damaged DNA Using Thiol-Tethered Oligonucleotide Duplexes
Source: PLoS One. 2015 Feb 13;10(2):e0117798. doi: 10.1371/journal.pone.0117798 (PMC4332495; doi:10.1371/journal.pone.0117798)

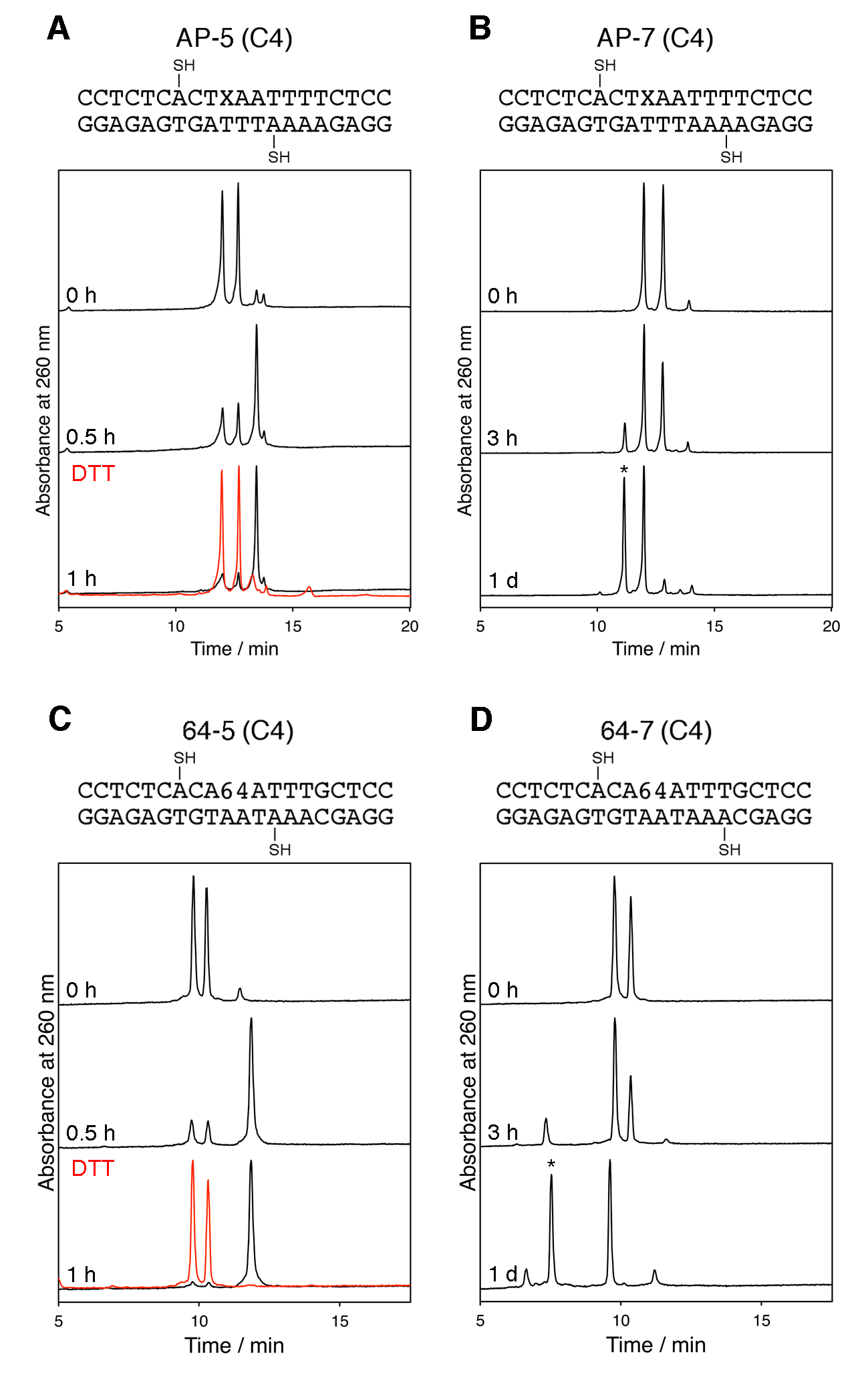

Supplement: S1 Fig — The y-axis of each chromatogram was normalized. The oligonucleotides containing sulfinic acid are indicated by an asterisk, and the results of the DTT treatment are shown in red. (TIF) [file pone.0117798.s001.tif]

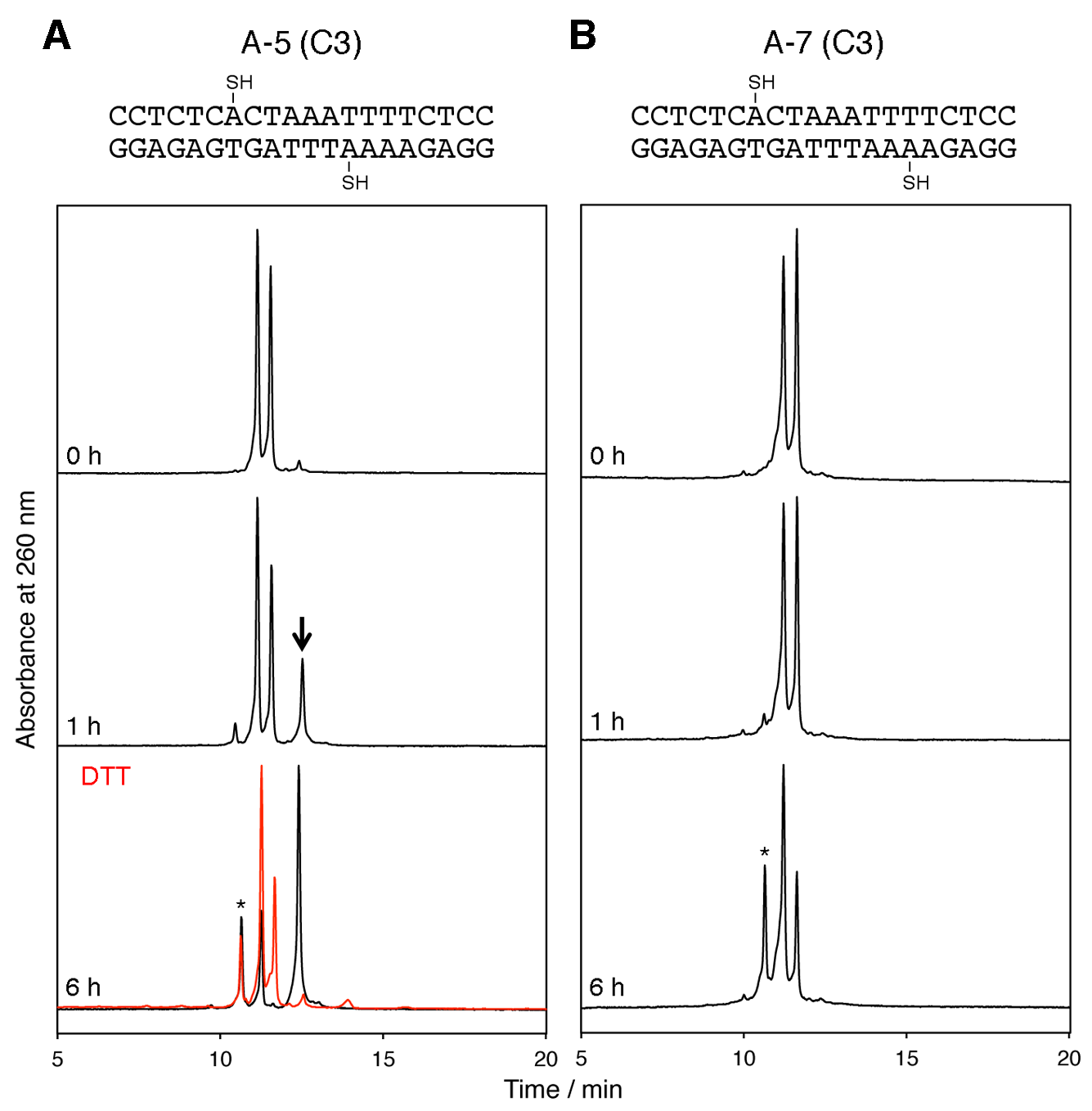

Supplement: S2 Fig — The y-axis of each chromatogram was normalized. The cross-linked products and the sulfinic acid-containing oligonucleotides are indicated by an arrow and an asterisk, respectively, and the result of the DTT treatment is shown in red. (TIF) [file pone.0117798.s002.tif]

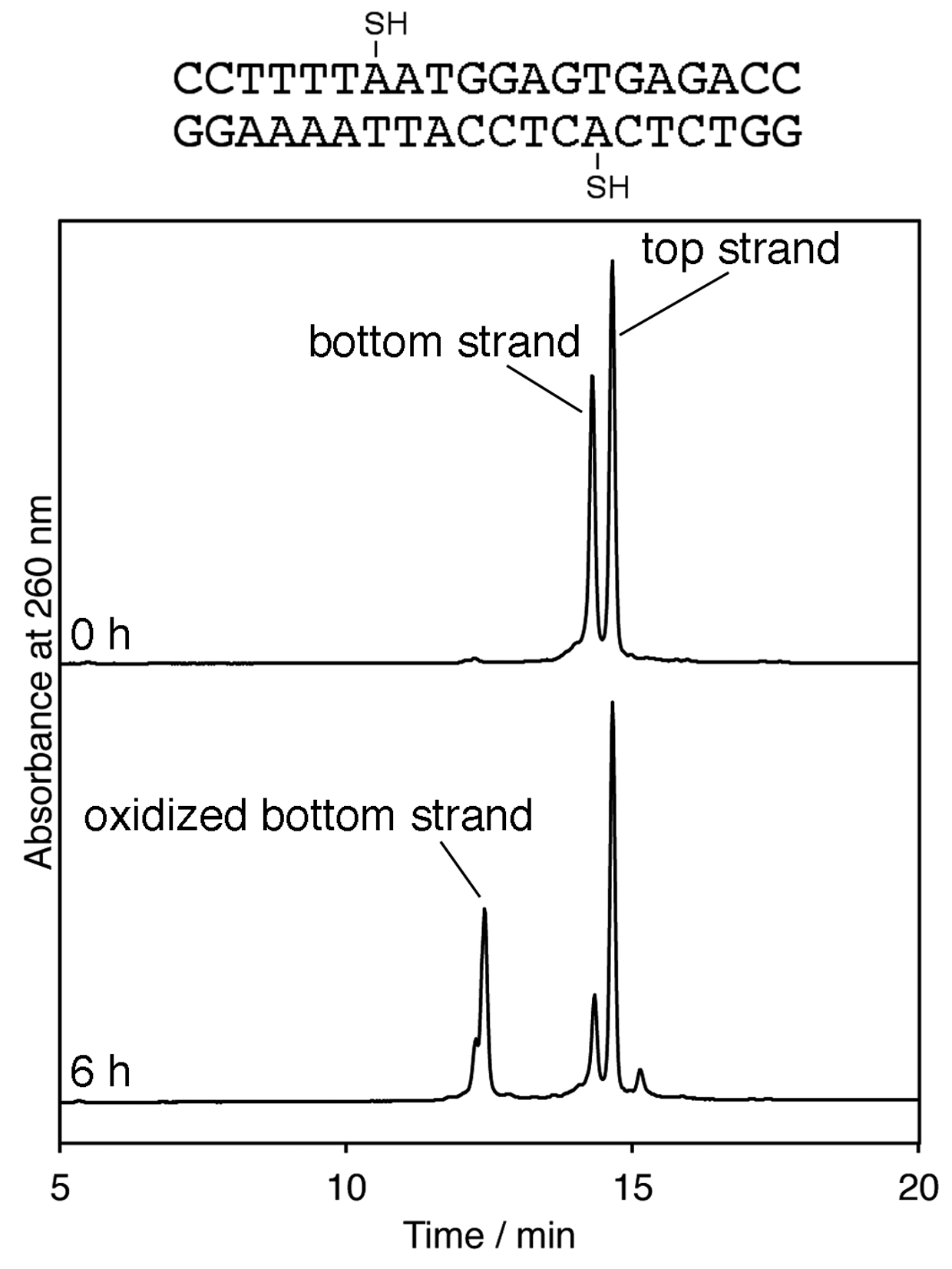

Supplement: S3 Fig — The peaks of the starting oligonucleotides were assigned by co-injection. The product had the same UV absorption spectrum as the bottom strand. (TIF) [file pone.0117798.s003.tif]
