# Supplementary material for: Analysis of Structural Flexibility of Damaged DNA Using Thiol-Tethered Oligonucleotide Duplexes
Source: PLoS One. 2015 Feb 13;10(2):e0117798. doi: 10.1371/journal.pone.0117798 (PMC4332495; doi:10.1371/journal.pone.0117798)
Supplement: S1 Protocol — (DOCX) [file pone.0117798.s004.docx]

Synthesis of 9-[2-*O*-[4-(tritylthio)butyl]-β-D-arabinofuranosyl]adenine (**2a**) and 9-[2-*O*-[3-(tritylthio)propyl]-β-D-arabinofuranosyl]adenine (**2b**)

A solution of triphenylmethanethiol (8.00 g, 28.9 mmol) in ethanol (30 ml) was mixed with 6% aqueous NaOH (20 ml), and the mixture was stirred for 15 min. To this mixture, a solution of 1,4-dibromobutane (5.15 ml, 43.4 mmol) in ethanol (20 ml) was added dropwise over 30 min. After stirring for 4 h, the precipitate was collected by filtration and dissolved in chloroform (60 ml). This solution was washed with water (60 ml) and with saturated aqueous NaCl (60 ml), and the organic layer was dried with sodium sulfate. The solvent was removed by evaporation, and hexane (50 ml) was added to the residue. After cooling at 4°C for 2 h, the precipitate was collected by filtration, and this product, 1-bromo-4-tritylthiobutane, was dried in a vacuum desiccator over phosphorus oxide. To a solution of 9-(β-D-arabinofuranosyl)adenine (**1**) (1.74 g, 6.51 mmol) in dimethylformamide (60 ml), sodium hydride (60% dispersion in mineral oil, 0.546 g, 13.7 mmol) was added at 0°C, and the mixture was stirred at 0°C for 1 h. To this mixture, a solution of 1-bromo-4-tritylthiobutane (3.15 g, 7.64 mmol) in dimethylformamide (60 ml) was added dropwise over 25 min, and the resultant mixture was stirred at room temperature for 3 h. Ice-cold water (30 ml) was added, and the product was extracted with chloroform (170 ml). After the organic layer was washed with water (170 ml) and with saturated aqueous NaCl (170 ml), methanol (25 ml) was added, and the insoluble material was removed by filtration. The solution was dried with sodium sulfate, concentrated *in vacuo*, and applied to a silica gel column (80 g). The elution was performed by a stepwise gradient of 0–7% methanol in chloroform, and two products (**2a** and the 5’ isomer) were eluted with 4–7% methanol. The desired product (**2a**) was partly separated by reversed-phase column chromatography, using Waters Preparative C18 125 Å 55–105 µm resin packed in a Bio-Rad Econo-Column (1.5 × 50 cm), with a linear gradient of acetonitrile (45–85% in water over 400 min) at a flow rate of 2.0 ml/min on a Bio-Rad BioLogic LP System. The product was obtained as a white foam after evaporation of the fractions, and was dried in a vacuum desiccator over phosphorus oxide. Yield: 1.06 g (1.78 mmol, 27%). In addition, 0.94 g (1.57 mmol) of a mixture of **2a** and the 5’ isomer was obtained. ^1^H NMR (400 MHz, DMSO-*d*_6_): δ = 8.12 (s, 1H; H2), 8.08 (s, 1H; H8), 7.31–7.19 (m, 17H; Tr, -NH_2_), 6.34 (d, *J* = 5.6 Hz, 1H; H1’), 5.55 (d, *J* = 5.1 Hz, 1H; 3’-OH), 4.97 (t, *J* = 5.1 Hz, 1H; 5’-OH), 4.21 (q, *J* = 5.7 Hz, 1H; H3’), 4.01 (t, *J* = 5.8 Hz, 1H; H2’), 3.73 (m, 1H; H4’), 3.64–3.58 (m, 2H; H5’), 3.26 (m, 1H; -OCH_2_-), 2.97 (q, *J* = 7.3 Hz, 1H; -OCH_2_-), 1.88 (t, *J* = 7.0 Hz, 2H; -CH_2_S-), 1.15–0.85 ppm (m, 4H; -OCH_2_C*H_2_*C*H_2_*CH_2_S-). ^13^C NMR (100.53 MHz, DMSO-*d*_6_): δ = 155.82, 152.38, 149.24, 144.44, 139.90, 128.97, 127.87, 126.52, 118.02, 83.59, 83.04, 81.51, 72.26, 69.36, 65.89, 60.33, 30.84, 28.29, 24.21 ppm. FAB-HRMS: *m/z* 598.2460 ([M+H]^+^; calcd for C_33_H_36_O_4_N_5_S, 598.2488).

The propyl counterpart (**2b**) was synthesized in the same manner. ^1^H NMR (500 MHz, DMSO-*d*_6_): δ = 8.10 (s, 1H; H2), 8.07 (s, 1H; H8), 7.32–7.20 (m, 17H; Tr, -NH_2_), 6.33 (d, *J* = 5.7 Hz, 1H; H1’), 5.55 (d, *J* = 5.2 Hz, 1H; 3’-OH), 4.96 (t, *J* = 5.6 Hz, 1H; 5’-OH), 4.20 (q, *J* = 5.8 Hz, 1H; H3’), 4.00 (t, *J* = 5.8 Hz, 1H; H2’), 3.71 (m, 1H; H4’), 3.61–3.56 (m, 2H; H5’), 3.31 (m, 1H; -OCH_2_-), 3.03 (dt, *J* = 9.6, 6.4 Hz, 1H; -OCH_2_-), 1.86 (m, 2H; -CH_2_S-), 1.15–1.12 ppm (m, 2H; -OCH_2_C*H_2_*CH_2_S-). ^13^C NMR (100.53 MHz, DMSO-*d*_6_): δ = 155.83, 152.40, 149.24, 144.35, 139.87, 128.95, 127.88, 126.52, 118.03, 83.54, 83.19, 81.52, 72.39, 68.68, 65.91, 60.41, 28.25, 27.67 ppm. FAB-HRMS: *m/z* 584.2310 ([M+H]^+^; calcd for C_32_H_34_O_4_N_5_S, 584.2332).
