# Supplementary material for: Analysis of Structural Flexibility of Damaged DNA Using Thiol-Tethered Oligonucleotide Duplexes
Source: PLoS One. 2015 Feb 13;10(2):e0117798. doi: 10.1371/journal.pone.0117798 (PMC4332495; doi:10.1371/journal.pone.0117798)
Supplement: S2 Protocol — (DOCX) [file pone.0117798.s005.docx]

Synthesis of 6-*N*-benzoyl-9-[2-*O*-[4-(tritylthio)butyl]-β-D-arabinofuranosyl]adenine (**3a**) and 6-*N*-benzoyl-9-[2-*O*-[3-(tritylthio)propyl]-β-D-arabinofuranosyl]adenine (**3b**)

To a solution of 9-[2-*O*-[4-(tritylthio)butyl]-β-D-arabinofuranosyl]adenine (**2a**) (1.06 g, 1.78 mmol) in pyridine (5.4 ml), benzoyl chloride (1.24 ml, 10.7 mmol) was added at 0°C, and the mixture was stirred at room temperature. After 1 h, saturated aqueous NaHCO_3_ (100 ml) was added, and the product was extracted with chloroform (330 ml in total). The organic layer was washed with saturated aqueous NaHCO_3_ (65 ml) and with saturated aqueous NaCl (65 ml), and was concentrated *in vacuo*. The residue was dissolved in a mixture of pyridine (5.4 ml) and tetrahydrofuran (3.6 ml), and a mixture of 2N NaOH (7.1 ml) and ethanol (7.1 ml) was added at 0°C. After 1.5 h at room temperature, the mixture was neutralized with 2 N HCl at 0°C, and saturated aqueous NaHCO_3_ (100 ml) was added. The product was extracted with chloroform (330 ml in total). The organic layer was washed with saturated aqueous NaHCO_3_ (65 ml) and with saturated aqueous NaCl (65 ml), and then dried with sodium sulfate and evaporated *in vacuo*. After co-evaporation with toluene, the residue was chromatographed on silica gel (60 g) with a step gradient of 0–4% methanol in chloroform. The appropriate fractions (2.0–3.5% methanol) were collected and concentrated. The product (**3a**) was obtained as a white foam, and was dried in a vacuum desiccator over phosphorus oxide. Yield: 1.23 g (1.76 mmol, 99%). ^1^H NMR (270 MHz, DMSO-*d*_6_): δ = 11.12 (s, 1H; -NH-), 8.67 (s, 1H; H8), 8.49 (s, 1H; H2), 8.02 (d, *J* = 7.3 Hz, 2H; Bz), 7.64 (t, *J* = 7.3 Hz, 1H; Bz), 7.53 (t, *J* = 7.4 Hz, 2H; Bz), 7.32–7.15 (m, 15H; Tr), 6.52 (d, *J* = 5.7 Hz, 1H; H1’), 5.62 (d, *J* = 5.1 Hz, 1H; 3’-OH), 5.01 (t, *J* = 5.4 Hz, 1H; 5’-OH), 4.27 (q, *J* = 6.0 Hz, 1H; H3’), 4.12 (t, *J* = 5.9 Hz, 1H; H2’), 3.79 (m, 1H; H4’), 3.70–3.59 (m, 2H; H5’), 3.30 (m, 1H; -OCH_2_-), 3.02 (m, 1H; -OCH_2_-), 1.88 (t, *J* = 7.0 Hz, 2H; -CH_2_S-), 1.18–0.83 ppm (m, 4H; -OCH_2_C*H_2_*C*H_2_*CH_2_S-). ^13^C NMR (100.53 MHz, DMSO-*d*_6_): δ = 165.36, 152.08, 151.43, 150.08, 144.45, 143.48, 133.38, 132.27, 128.97, 128.39, 128.33, 127.84, 126.48, 125.23, 83.56, 83.27, 81.79, 72.13, 69.53, 65.88, 60.23, 30.85, 28.28, 24.17 ppm. FAB-HRMS: *m/z* 702.2773 ([M+H]^+^; calcd for C_40_H_40_O_5_N_5_S, 702.2750).

The propyl counterpart (**3b**) was synthesized in the same manner. ^1^H NMR (400 MHz, DMSO-*d*_6_): δ = 11.12 (s, 1H; -NH-), 8.68 (s, 1H; H8), 8.43 (s, 1H; H2), 8.04 (d, *J* = 7.4 Hz, 2H; Bz), 7.64 (t, *J* = 7.4 Hz, 1H; Bz), 7.54 (t, *J* = 7.5 Hz, 2H; Bz), 7.30–7.16 (m, 15H; Tr), 6.49 (d, *J* = 5.8 Hz, 1H; H1’), 5.60 (d, *J* = 5.2 Hz, 1H; 3’-OH), 4.98 (t, *J* = 5.5 Hz, 1H; 5’-OH), 4.25 (q, *J* = 5.8 Hz, 1H; H3’), 4.10 (t, *J* = 5.8 Hz, 1H; H2’), 3.76 (m, 1H; H4’), 3.66–3.55 (m, 2H; H5’), 3.36 (dt, *J* = 9.6, 6.4 Hz, 1H; -OCH_2_-), 3.09 (dt, *J* = 9.6, 6.4 Hz, 1H; -OCH_2_-), 1.89 (m, 2H; -CH_2_S-), 1.15 ppm (q, *J* = 6.8 Hz, 2H; -OCH_2_C*H_2_*CH_2_S-). ^13^C NMR (100.53 MHz, DMSO-*d*_6_): δ = 165.42, 152.09, 151.47, 150.05, 144.39, 143.46, 133.39, 132.30, 128.95, 128.42, 128.36, 127.86, 126.51, 124.64, 83.43, 81.69, 79.11, 72.26, 68.79, 65.93, 60.29, 28.21, 27.70 ppm. FAB-HRMS: *m/z* 688.2598 ([M+H]^+^; calcd for C_39_H_38_O_5_N_5_S, 688.2594).
