# Supplementary material for: Analysis of Structural Flexibility of Damaged DNA Using Thiol-Tethered Oligonucleotide Duplexes
Source: PLoS One. 2015 Feb 13;10(2):e0117798. doi: 10.1371/journal.pone.0117798 (PMC4332495; doi:10.1371/journal.pone.0117798)
Supplement: S3 Protocol — (DOCX) [file pone.0117798.s006.docx]

Synthesis of 6-*N*-benzoyl-9-[5-*O*-(4,4'-dimethoxytrityl)-2-*O*-[4-(tritylthio)butyl]-β-D-arabinofuranosyl]adenine (**4a**) and 6-*N*-benzoyl-9-[5-*O*-(4,4'-dimethoxytrityl)-2-*O*-[3-(tritylthio)propyl]-β-D-arabinofuranosyl]adenine (**4b**)

After dehydration by co-evaporation with pyridine, 6-*N*-benzoyl-9-[2-*O*-[4-(tritylthio)butyl]-β-D-arabinofuranosyl]adenine (**3a**) (1.39 g, 1.98 mmol) was dissolved in pyridine (8 ml), and 4,4’-dimethoxytrityl chloride (1.03 g, 3.04 mmol) was added to this solution. The mixture was stirred at room temperature for 1 h. Methanol (4 ml) was added, and the solution was concentrated *in vacuo*. The residue was dissolved in chloroform (310 ml), and the solution was washed with saturated aqueous NaHCO_3_ (170 ml) and with saturated aqueous NaCl (170 ml), and then dried with sodium sulfate and evaporated *in vacuo*. After co-evaporation with toluene, the residue was chromatographed on silica gel (50 g) with a step gradient of 0–2% methanol in chloroform. The appropriate fractions (0.5–1.5% methanol) were collected and concentrated. The product (**4a**) was obtained as a white foam, and was dried in a vacuum desiccator over phosphorus oxide. Yield: 1.61 g (1.61 mmol, 81%). ^1^H NMR (270 MHz, DMSO-*d*_6_): δ = 11.14 (s, 1H; -NH-), 8.67 (s, 1H; H8), 8.25 (s, 1H; H2), 8.02 (d, *J* = 7.2 Hz, 2H; Bz), 7.64 (t, *J* = 7.3 Hz, 1H; Bz), 7.53 (t, *J* = 7.4 Hz, 2H; Bz), 7.36 (d, *J* = 7.0 Hz, 2H; DMT), 7.32–7.18 (m, 22H; Tr, DMT), 6.81 (dd, *J* = 5.6, 8.8 Hz, 4H; DMT), 6.55 (d, *J* = 5.3 Hz, 1H; H1’), 5.68 (d, *J* = 5.2 Hz, 1H; 3’-OH), 4.25 (q, *J* = 4.9 Hz, 1H; H3’), 4.05 (m, 2H; H2’, H4’), 3.69 (d, 6H; -OCH_3_), 3.35 (m, 1H; -OCH_2_-), 3.20 (m, 2H; H5’), 2.97 (m, 1H; -OCH_2_-), 1.87 (t, *J* = 7.0 Hz, 2H; -CH_2_S-), 1.14–0.85 ppm (m, 4H; -OCH_2_C*H_2_*C*H_2_*CH_2_S-). ^13^C NMR (100.53 MHz, DMSO-*d*_6_): δ = 165.39, 157.97, 151.98, 151.50, 150.18, 144.65, 144.44, 143.33, 135.56, 133.42, 132.28, 129.58, 128.96, 128.40, 128.32, 127.83, 127.65, 126.58, 126.48, 124.73, 113.04, 85.34, 83.12, 82.15, 79.11, 73.41, 69.27, 65.86, 63.59, 54.94, 30.77, 28.18, 24.16 ppm. FAB-HRMS: *m/z* 1004.4031 ([M+H]^+^; calcd for C_61_H_58_O_7_N_5_S, 1004.4057).

The propyl counterpart (**4b**) was synthesized in the same manner. ^1^H NMR (400 MHz, DMSO-*d*_6_): δ = 11.13 (s, 1H; -NH-), 8.67 (s, 1H; H8), 8.19 (s, 1H; H2), 8.04 (d, *J* = 7.3 Hz, 2H; Bz), 7.64 (t, *J* = 7.4 Hz, 1H; Bz), 7.54 (t, *J* = 7.6 Hz, 2H; Bz), 7.36 (d, *J* = 7.1 Hz, 2H; DMT), 7.27–7.14 (m, 22H; Tr, DMT), 6.81 (t, *J* = 9.0 Hz, 4H; DMT), 6.52 (d, *J* = 5.5 Hz, 1H; H1’), 5.66 (d, *J* = 5.2 Hz, 1H; 3’-OH), 4.24 (q, *J* = 5.4 Hz, 1H; H3’), 4.05 (t, *J* = 5.3 Hz, 1H; H2’), 3.98 (td, *J* = 6.3, 3.1 Hz, 1H; H4’), 3.68 (d, 6H; -OCH_3_), 3.32 (m, 1H; -OCH_2_-), 3.18 (dd, *J* = 10.2, 3.2 Hz, 2H; H5’), 3.02 (dt, *J* = 9.6, 6.4 Hz, 1H; -OCH_2_-), 1.85 (m, 2H; -CH_2_S-), 1.10 ppm (q, *J* = 6.7 Hz, 2H; -OCH_2_C*H_2_*CH_2_S-). ^13^C NMR (100.53 MHz, DMSO-*d*_6_): δ = 165.44, 157.98, 151.98, 151.53, 150.18, 144.68, 144.32, 143.26, 135.53, 133.44, 132.30, 129.60, 128.94, 128.41, 128.35, 127.83, 127.63, 126.58, 126.48, 124.68, 113.04, 85.36, 83.13, 81.98, 79.12, 73.55, 68.59, 65.93, 63.51, 54.95, 28.11, 27.63 ppm. FAB-HRMS: *m/z* 990.3956 ([M+H]^+^; calcd for C_60_H_56_O_7_N_5_S, 990.3900).
