# Supplementary material for: Analysis of Structural Flexibility of Damaged DNA Using Thiol-Tethered Oligonucleotide Duplexes
Source: PLoS One. 2015 Feb 13;10(2):e0117798. doi: 10.1371/journal.pone.0117798 (PMC4332495; doi:10.1371/journal.pone.0117798)
Supplement: S4 Protocol — (DOCX) [file pone.0117798.s007.docx]

Synthesis of 6-*N*-benzoyl-9-[5-*O*-(4,4'-dimethoxytrityl)-3-*O*-[[bis(1-methylethyl)amino](2-cyanoethoxy)phosphino]-2-*O*-[4-(tritylthio)butyl]-β-D-arabinofuranosyl]adenine (**5a**) and 6-*N*-benzoyl-9-[5-*O*-(4,4'-dimethoxytrityl)-3-*O*-[[bis(1-methylethyl)amino](2-cyanoethoxy)phosphino]-2-*O*-[3-(tritylthio)propyl]-β-D-arabinofuranosyl]adenine (**5b**)

To a solution of 6-*N*-benzoyl-9-[5-*O*-(4,4'-dimethoxytrityl)-2-*O*-[4-(tritylthio)butyl]-β-D-arabinofuranosyl]adenine (**4a**) (557 mg, 555 µmol) in tetrahydrofuran (4.7 ml), *N*,*N*-diisopropylethylamine (374 µl, 2.20 mmol) and 2-cyanoethyl *N*,*N*-diisopropylchlorophosphoramidite (246 µl, 1.10 mmol) were added. The mixture was stirred for 1 h, and diluted with ethyl acetate (130 ml). This solution was washed with 2% aqueous NaHCO_3_ (130 ml) and with water (130 ml), dried with sodium sulfate, concentrated to a gum, and co-evaporated with toluene. The residue was chromatographed on silica gel (14 g), with a step gradient of 25–70% ethyl acetate in hexane containing 0.5% pyridine. The appropriate fractions (40–55% ethyl acetate) were collected, concentrated, and co-evaporated with acetonitrile. The product (**5a**) was obtained as a foam, and was dried in a vacuum desiccator over phosphorus oxide. Yield: 510 mg (424 µmol, 76%). ^1^H NMR (500 MHz, acetone-*d*_6_): δ = 9.83 (s, 1H; -NH-), 8.62 (d, *J* = 5.1 Hz, 1H; H8), 8.25 (d, *J* = 8.5 Hz, 1H; H2), 8.08 (d, *J* = 7.3 Hz, 2H; Bz), 7.64 (t, *J* = 7.2 Hz, 1H; Bz), 7.54 (t, *J* = 7.8 Hz, 2H; Bz), 7.50 (m, 2H; DMT), 7.40–7.18 (m, 22H; Tr, DMT), 6.85 (m, 4H; DMT), 6.65 (d, *J* = 4.7 Hz, 1H; H1’), 4.67 (m, 1H; H3’), 4.30 (m, 1H; H2’), 4.25 (m, 1H; H4’), 3.83–3.68 (m, 10H; -C*H*_2_CH_2_CN, -OCH_3_, H5’), 3.52–3.07 (m, 4H; -CH_2_C*H*_2_CN, -C*H*(CH_3_)_2_), 2.74 (m, 1H; -OCH_2_-), 2.65 (t, *J* = 6.1 Hz, 1H; -OCH_2_-), 1.97 (m, 2H; -CH_2_S-), 1.27–1.02 ppm (m, 16H; -OCH_2_C*H_2_*C*H_2_*CH_2_S-, -CH(C*H_3_*)_2_). ^13^C NMR (100.53 MHz, acetone-*d*_6_): δ = 165.83, 159.90, 153.36, 153.02, 146.28, 146.24, 144.41, 144.33, 137.00, 135.41, 133.38, 131.24, 130.62, 129.64, 129.35, 129.28, 128.93, 128.84, 127.86, 127.66, 125.20, 119.10, 114.19, 87.25, 84.75, 84.54, 84.30, 77.43, 71.25, 67.44, 64.65, 59.94, 55.81, 44.37, 32.37, 25.84, 25.20, 23.40, 20.99 ppm. ^31^P NMR (202.07 MHz, acetone-*d*_6_): δ = 147.23 ppm. FAB-HRMS: *m/z* 1204.5115 ([M+H]^+^; calcd for C_70_H_75_O_8_N_7_PS, 1204.5135).

The propyl counterpart (**5b**) was synthesized in the same manner. ^1^H NMR (400 MHz, acetone-*d*_6_): δ = 9.82 (s, 1H; -NH-), 8.62 (d, *J* = 4.4 Hz, 1H; H8), 8.17 (d, *J* = 8.7 Hz, 1H; H2), 8.08 (d, *J* = 7.5 Hz, 2H; Bz), 7.63 (t, *J* = 7.3 Hz, 1H; Bz), 7.54 (t, *J* = 7.7 Hz, 2H; Bz), 7.50 (m, 2H; DMT), 7.37–7.15 (m, 22H; Tr, DMT), 6.84 (m, 4H; DMT), 6.62 (d, *J* = 4.7 Hz, 1H; H1’), 4.65 (m, 1H; H3’), 4.29 (m, 1H; H2’), 4.22 (m, 1H; H4’), 3.84–3.65 (m, 10H; -C*H*_2_CH_2_CN, -OCH_3_, H5’), 3.49–3.11 (m, 4H; -CH_2_C*H*_2_CN, -C*H*(CH_3_)_2_), 2.75 (m, 1H; -OCH_2_-), 2.63 (t, *J* = 6.3 Hz, 1H; -OCH_2_-), 1.96 (m, 2H; -CH_2_S-), 1.34–1.05 ppm (m, 14H; -OCH_2_C*H_2_*CH_2_S-, -CH(C*H_3_*)_2_). ^13^C NMR (100.53 MHz, acetone-*d*_6_): δ = 168.48, 159.92, 153.31, 153.05, 146.29, 146.09, 144.35, 144.27, 136.99, 135.47, 133.41, 131.26, 130.60, 129.67, 129.34, 129.27, 128.94, 128.85, 127.87, 127.68, 125.07, 119.08, 114.19, 87.26, 84.59, 84.39, 84.06, 77.45, 70.47, 67.58, 64.51, 59.95, 55.82, 44.36, 29.23, 25.20, 23.35, 21.03 ppm. ^31^P NMR (161.83 MHz, acetone-*d*_6_): δ = 147.25, 147.13 ppm. FAB-HRMS: *m/z* 1190.5000 ([M+H]^+^; calcd for C_69_H_73_O_8_N_7_PS, 1190.4979).
